# Association Between the 2022 AHA/ACC/HFSA Heart Failure Staging and Cardiovascular and Kidney Outcomes in Patients With Diabetes and Kidney Disease: A Post Hoc Analysis of the SCORED Randomized Controlled Trial

**DOI:** 10.1161/CIRCHEARTFAILURE.125.013054

**Published:** 2026-01-22

**Authors:** Ayodele Odutayo, Deepak L. Bhatt, Vikas S. Sridhar, Michael Szarek, Christopher P. Cannon, Lawrence A. Leiter, Darren K. McGuire, Julia B. Lewis, Renato D. Lopes, Benjamin M. Scirica, Kausik K. Ray, Michael J. Davies, Phillip Banks, Manon Girard, Subodh Verma, Jacob A. Udell, Bertram Pitt, Ph. Gabriel Steg, David Z.I. Cherney

**Affiliations:** 1 Sunnybrook Research Institute, Sunnybrook Health Sciences Centre, Toronto, ON, Canada (A.O.).; 2 University Health Network (A.O., V.S.S., J.A.U., D.Z.I.C.), University of Toronto, ON, Canada.; 3 Women’s College Hospital (J.A.U.), University of Toronto, ON, Canada.; 4 Peter Munk Cardiac Centre, University Health Network (J.A.U.), University of Toronto, ON, Canada.; 5 University of Toronto, ON, Canada (A.O., V.S.S., L.A.L., S.V., D.Z.I.C.).; 6 Mount Sinai Fuster Heart Hospital, Icahn School of Medicine at Mount Sinai, New York, NY (D.L.B.).; 7 University of Colorado Anschutz Medical Campus, Aurora (M.S.).; 8 Division of Cardiovascular Medicine, Brigham and Women’s Hospital, Harvard Medical School, Boston, MA (C.P.C., B.M.S.).; 9 Li Ka Shing Knowledge Institute, St. Michael’s Hospital, Toronto, ON, Canada (L.A.L., S.V.).; 10 University of Texas Southwestern Medical Center and Parkland Health and Hospital System, Dallas (D.K.M.).; 11 Vanderbilt University Medical Center, Nashville, TN (J.B.L.).; 12 Duke Clinical Research Institute, Duke University School of Medicine, Durham, NC (R.D.L.).; 13 Department of Primary Care and Public Health, Imperial Centre for Cardiovascular Disease Prevention, Imperial College London, United Kingdom (K.K.R.).; 14 Lexicon Pharmaceuticals Inc, The Woodlands, TX (M.J.D., P.B., M.G.).; 15 University of Michigan, Ann Arbor (B.P.).; 16 Assistance Publique-Hôpitaux de Paris, Hôpital Bichat, INSERM U-1148, Université Paris-Cité, France (P.G.S.).

**Keywords:** biomarkers, coronary artery disease, heart failure, hypertrophy, renal insufficiency

## Abstract

**BACKGROUND:**

The 2022 American Heart Association/American College of Cardiology/Heart Failure Society of America heart failure (HF) classification incorporates cardiac biomarkers to identify early risk of HF. The HF stages may also guide the prognosis and management of cardiovascular and kidney-related events.

**METHODS:**

SCORED (Effect of Sotagliflozin on Cardiovascular and Renal Events in Patients With Type 2 Diabetes and Moderate Renal Impairment Who Are at Cardiovascular Risk) was a randomized trial in diabetes with kidney disease comparing sotagliflozin versus placebo on cardiovascular death, HF hospitalizations, and urgent HF visits. SCORED participants were grouped by HF stage post hoc. Stage A: no HF, normal biomarkers (NT-proBNP [N-terminal pro-B-type natriuretic peptide] <125 pg/mL; high sensitivity cardiac troponin T [hs-cTnT] <14 ng/L), and normal cardiac structure/function. Stage B (pre-HF): no HF but elevated NT-proBNP, hs-cTnT, or abnormal cardiac structure/function. Stage C/D: symptomatic HF. End points include the primary composite (cardiovascular death and HF-related events), major adverse cardiovascular events, and kidney-related composites (≥50% decline in estimated glomerular filtration rate, kidney failure, or kidney death). Using competing-risk proportional hazards models, we examined the association between HF stage and these end points, and the effect of sotagliflozin versus placebo by HF stage.

**RESULTS:**

There were 741 patients (7%) in stage A, 6560 (62%) in stage B (pre-HF), and 3283 (31%) in stage C/D (established HF). The median NT-proBNP and hs-cTnT increased with HF stage. Increasing HF stage was associated with a 2- to 4-fold increase in the primary outcome/major adverse cardiovascular events in the placebo group. The kidney-specific composite was 5-fold higher in stage B (pre-HF) versus stage A but similar in stages B and C/D. The effect of sotagliflozin versus placebo was similar, irrespective of HF stage (primary outcome: hazard ratio, 0.74 [95% CI, 0.63–0.88]; *P*_interaction_=1.00), with higher absolute benefit in each HF stage (*P*-trend_IRR_=0.002). The absolute benefit for the kidney-specific end point was comparable for stages B and C/D.

**CONCLUSIONS:**

Increasing HF stage is associated with a higher risk of HF, major adverse cardiovascular events, and kidney events. Asymptomatic stage B (pre-HF) increased cardiovascular and renal events by >2- and 5-fold, respectively. The benefits of sotagliflozin are consistent, irrespective of HF stage.

**What is New?**

- Increasing heart failure (HF) stage is associated with a higher risk of HF, major adverse cardiovascular events, and kidney events.
- Asymptomatic HF stage B (pre-HF) is associated with >2- and 5-fold increased cardiovascular and kidney risk, respectively.
- The benefits of sotagliflozin are consistent, irrespective of HF stage.

**What Are the Clinical Implications?**

- The risk associated with HF stage B (pre-HF) should encourage physicians to stage HF risk among all patients with risk factors for HF, including hypertension, diabetes, and chronic kidney disease.
- Appropriate patients at risk for HF should be evaluated for SGLT (sodium-glucose cotransporter) inhibitor use.

**See Editorial by Bansal**

Diabetes and kidney disease are associated with an increased risk of heart failure (HF).^1^ Furthermore, incident HF reciprocally contributes to the progression of chronic kidney disease (CKD), resulting in a cycle of interdependent and progressive decline in heart and kidney function.^2^ Disrupting this cycle requires early identification and risk stratification of individuals at risk for HF and CKD, and initiation of kidney and cardioprotective medications such as SGLT (sodium-glucose cotransporter) inhibitors.^3–5^

The 2022 American Heart Association (AHA)/American College of Cardiology (ACC)/Heart Failure Society of America HF classification newly incorporates cardiac biomarkers to identify individuals at risk of HF before the development of overt symptoms, thereby identifying a patient population eligible for primary prevention of HF.^6^ The 2022 HF classification includes 4 categories. Stage A includes those without symptomatic HF who have normal cardiac biomarkers and normal cardiac structure/function. Stage B, also known as pre-HF, includes those without symptomatic HF but abnormal cardiac structure/function, elevated NT-proBNP (N-terminal pro-B-type natriuretic peptide), or hs-cTnT. Stages C and D include individuals with symptomatic HF. Given shared risk factors between HF and CKD, as well as the association between cardiac biomarkers and cardiorenal outcomes,^7,8^ the 2022 HF stages may inform risk stratification and management of cardiovascular and kidney outcomes beyond HF.

SCORED (Effect of Sotagliflozin on Cardiovascular and Renal Events in Patients With Type 2 Diabetes and Moderate Renal Impairment Who Are at Cardiovascular Risk) was a randomized controlled trial in adults with type 2 diabetes and CKD that examined the effect of sotagliflozin, a dual SGLT1/2 inhibitor, versus placebo on cardiovascular death, hospitalizations for HF, and urgent HF visits.^9–11^ We conducted a post hoc analysis of the SCORED trial to assess (1) how many participants are classified in each stage on the basis of the 2022 HF criteria, (2) the association between the 2022 HF staging and cardiovascular and kidney outcomes, and (3) whether the effect of sotagliflozin versus placebo varied by HF stage.

## Methods

SCORED was a multinational, double-blind randomized controlled trial comparing sotagliflozin to placebo in 10 584 patients with type 2 diabetes and CKD, defined as an estimated glomerular filtration rate (eGFR) of 25 to 60 mL/min per 1.73 m^2^, irrespective of urinary albumin-to-creatinine ratio (UACR), and either 1 major cardiovascular risk factor or age ≥55 years and at least 2 minor cardiovascular risk factors. Participants were recruited in 750 sites in 44 countries and randomized 1:1 to sotagliflozin (initially at 200 mg/d and subsequently increased to 400 mg/d) versus a matching placebo. The first participant was randomized on December 8, 2017, and the last on January 20, 2020. The study protocol and statistical analysis plan are reported with the primary publication.^9^ Ethics approval was obtained at all participating sites, and participants provided written informed consent. Data may be shared on a reasonable request to the corresponding author.

### Applying the 2022 HF Classification

Participants were classified using the 2022 AHA/ACC/Heart Failure Society of America HF classification.^6^ The 2022 classification categorizes participants into 4 stages of HF: stages A, B, C, and D.^6^ The 2022 classification now incorporates the use of biomarkers to classify participants as stage A or B.^6^ Stage A is defined as individuals with risk factors for HF but who do not have structural heart disease and have normal cardiac biomarkers (NT-proBNP <125 pg/mL and cardiac troponin).^6^ The 2022 AHA/ACC/HSFA HF classification does not specify a cutoff for hs-cTnT, and we selected <14 ng/L as this is the 99th percentile cutoff for the hs-cTnT assay used in SCORED. Risk factors include hypertension, coronary artery disease, diabetes, obesity, exposure to cardiotoxic agents, genetic variants for cardiomyopathy, and family history of cardiomyopathy.^6^ Stage B includes individuals with evidence of structural heart disease (reduced left or right ventricular systolic function, ventricular hypertrophy, chamber enlargement, wall motion abnormalities, and valvular heart disease) or evidence for elevated filling pressures by invasive or noninvasive techniques or elevated cardiac biomarkers (NT-proBNP ≥125 pg/mL or hs-cTnT ≥14 ng/L).^6^ Finally, stage C includes individuals with structural heart disease with current or previous symptoms of HF, and stage D includes individuals with marked HF symptoms that interfere with their daily life and with recurrent hospitalization despite attempts to optimize guideline-directed medical treatment.^6^

SCORED participants were stratified into HF stages using the 2022 classification system based on baseline comorbidities, cardiac biomarkers, and diagnostic findings. For baseline comorbidities, all SCORED participants had a detailed history and physical examination at the start of the trial. For cardiac biomarkers, participants completed blood work for NT-proBNP and hs-cTNT at baseline. NT-proBNP was measured using an electrochemiluminescent immunoassay on a Cobas analyzer (Roche Diagnostics). hs-cTnT was measured using an electrochemiluminescent immunoassay (Troponin T Gen 5 STAT, Roche Diagnostics).

Finally, with respect to diagnostic test findings, all participants had an assessment of left ventricular ejection fraction (LVEF) within the year before study enrollment by either echocardiogram, multiple gated acquisition scan, magnetic resonance imaging, positron emission tomography, single-photon emission computed tomography, or left ventricular angiography. As well, participants had left ventricular hypertrophy assessed based on ECG or echocardiography. SCORED did not include information on other detailed echocardiographic parameters for structural heart disease or elevated filling pressures. Specific to SCORED, all participants had diabetes and CKD and were, therefore, classified as at least stage A or higher. Participants with elevated biomarkers (NT-proBNP ≥125 pg/mL or hs-cTnT ≥14 ng/L), LVEF<50%, or evidence of left ventricular hypertrophy, in the absence of symptoms of HF, were classified as stage B. Finally, participants with an established diagnosis of HF at baseline were classified as stage C/D. We grouped participants in stages C and D together as SCORED did not have sufficient data on baseline HF symptoms to distinguish symptomatic and end-stage HF. Furthermore, SCORED excluded participants with New York Heart Association IV symptoms. This approach is similar to other studies of the 2022 AHA/ACC/Heart Failure Society of America HF stages.^12^

### Study Outcomes

The primary outcome of SCORED and the main outcome for this analysis were total occurrences after randomization of hospitalization for HF, urgent visit for HF, and death from cardiovascular causes.^9^ Additional outcomes examined included total major cardiovascular events (MACE), first CKD progression event, and first event in a cardiorenal composite. MACE was defined as the total number of events in the composite of cardiovascular death, nonfatal myocardial infarction, and nonfatal stroke. CKD progression was the prespecified kidney outcome in the SCORED primary publication and was a composite of ≥50% decline in eGFR (sustained or last value), kidney failure, or kidney death. For kidney outcomes, we included laboratory data to derive the eGFR components and case report form data for the nonlaboratory-defined components (eg, kidney transplant).^13^ The cardiorenal composite outcome was a combination of cardiovascular death and the CKD progression composite outcome.

### Statistical Analysis

Baseline participant characteristics were summarized using counts and percentages or medians and interquartile ranges. Incidence rates of outcomes were estimated as the number of events per 100 person-years of follow-up. Relative risk of events within the placebo group, quantified by hazard ratios (HRs) and associated 2-sided 95% CIs, were estimated across HF stages using multivariable competing-risk proportional hazards models (marginal models for total event end points), stratified by the randomization stratification factors (geographic region and HF criteria [LVEF ≤40% documented within the past year or hospitalization for HF during the previous 2 years]), and adjusted for potential baseline confounders (age, sex, race, body mass index, systolic blood pressure, diastolic blood pressure, duration since diabetes diagnosis, hemoglobin A1c, eGFR, UACR, history of cardiovascular disease [myocardial infarction, stroke, coronary revascularization, or peripheral vascular disease], and concomitant medications [renin-angiotensin-aldosterone system inhibitor, loop diuretic, β-blocker, statin, and antiplatelet]). Stage A served as the reference category, and tests of linear trend across HF stages represented interaction tests that accounted for the ordinal nature of the stages. However, as stage B was the largest subgroup of participants, we repeated our analysis with stage B as the reference. Deaths were treated as competing terminal events if not a component of a given end point, and a robust sandwich variance estimate for the SE of the log HR was applied to reflect the dependence of event times within individual patients for total event end points.

Efficacy analyses comparing sotagliflozin to placebo were performed according to the intention-to-treat principle. We used the competing-risk proportional hazards models described above, stratified by the randomization stratification factors, to assess the relative effects of sotagliflozin versus placebo. Treatment group, 2022 HF stage, and their interaction were included in the model, with treatment effects within each stage summarized by treatment HRs and associated 2-sided 95% CIs. Linear trend tests across HF stages assessed potential heterogeneity in the relative treatment effect of sotagliflozin versus placebo. Absolute risk reductions were derived as the differences in the incidence rates between sotagliflozin and placebo-treated groups and were compared across 2022 HF stages using tests for trend from Poisson regression models. All analyses were performed using SAS statistical software.

## Results

All 10 584 participants in SCORED were included in this analysis. Of these, 8 participants had missing data on at least 1 cardiac biomarker, 2 were missing LVEF measurements, and 43 were missing documentation of left ventricular hypertrophy (Table S1). These participants were all classified as HF stage A based on their available data.

Using the 2022 classification, there were 741 participants (7%) in stage A, 6560 (62%) in stage B, and 3283 (31%) in stage C/D. The baseline characteristics of participants are reported in the Table and Table S2. The percentage of women was higher in stage A versus stages B and C/D. As well, the median LVEF was 63% and 60% in stages A and B, respectively, but 48% in stage C/D. The median UACR and eGFR were comparable across stages, and the median NT-proBNP and hs-cTnT increased with HF stage (Table).

**Table.**
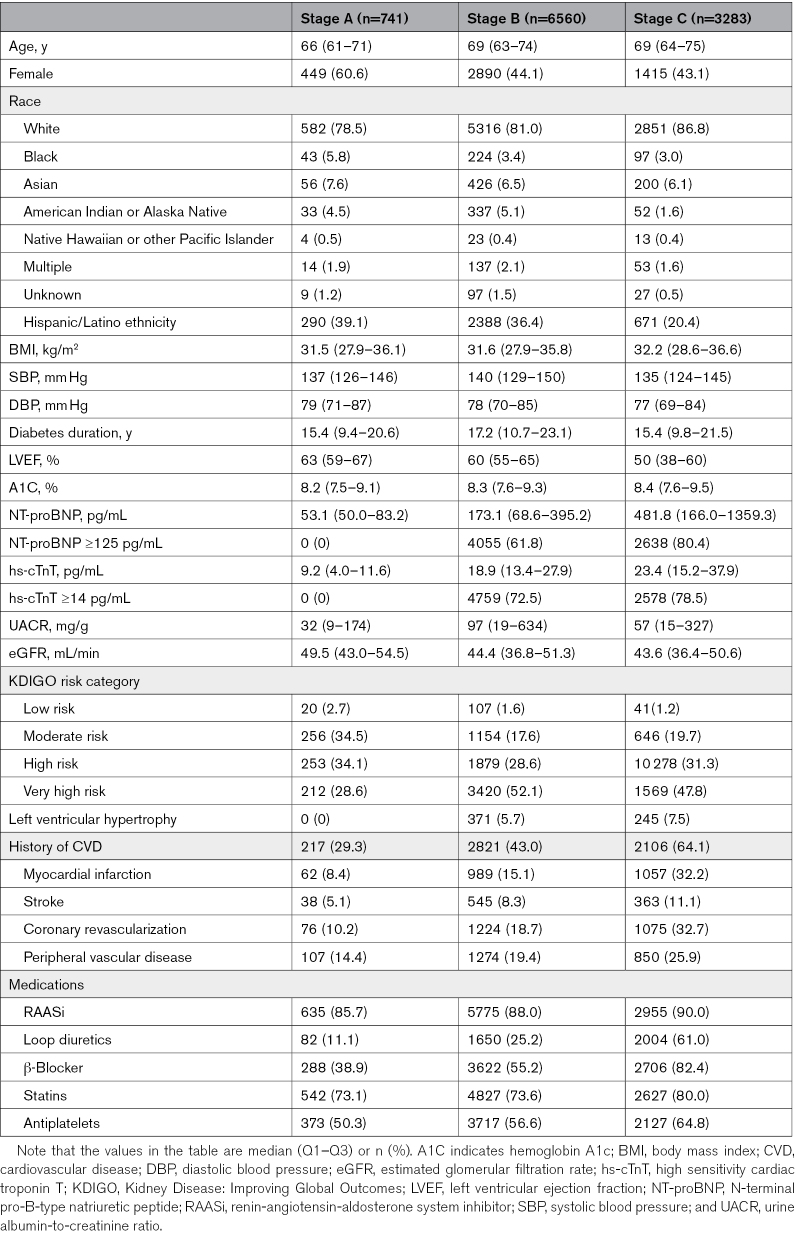
Baseline Characteristics of Participants by Heart Failure Stage Using the 2022 Classification

### Association Between the 2022 HF Stage and HF, MACE, and Kidney Outcomes

**Figure 1.**
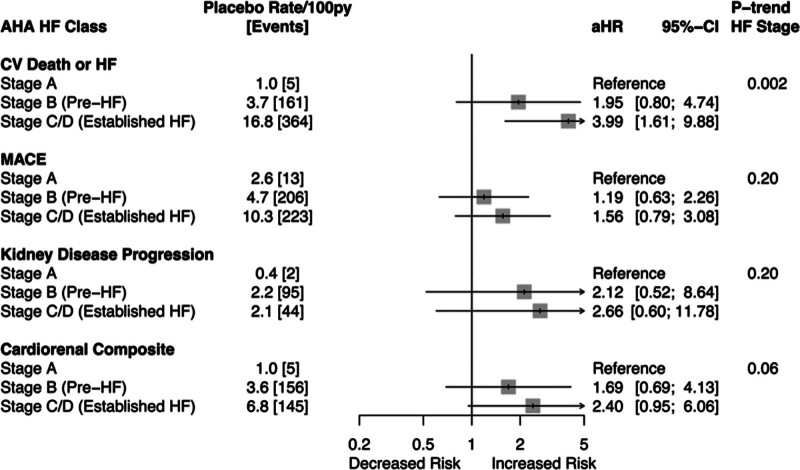
**Association of the 2022 heart failure (HF) stage with clinical outcomes.** MACE is a composite of cardiovascular (CV) death, nonfatal myocardial infarction, and nonfatal stroke. Kidney disease progression is a composite of ≥50% decline in estimated glomerular filtration rate (eGFR; sustained or last value), kidney failure, or kidney death. Cardiorenal composite includes ≥50% decline in eGFR (sustained or last value), kidney failure, CV death, or kidney death. All hazard ratios are adjusted for age, sex, race, body mass index, systolic blood pressure, diastolic blood pressure, diabetes duration, HbA1c, eGFR, urine albumin-to-creatinine ratio, history of CV disease (MI, stroke, coronary revascularization, or PVD), and concomitant medications (renin-angiotensin-aldosterone system inhibitor, loop diuretic, β-blocker, statin, and antiplatelet). AHA indicates American Heart Association; aHR, adjusted hazard ratio; HbA1c, hemoglobin A1c; MACE, major cardiovascular events; MI, myocardial infarction; and PVD, peripheral vascular disease; py, person-years.

Among participants in the placebo group, increasing HF stage was associated with a 2- to 4-fold increased incidence of the primary outcome (HF stage B versus stage A: HR, 1.95 [95% CI, 0.80–4.73]; HF stage C/D versus stage A: HR, 3.99 [95% CI, 1.61–9.87]; *P*=0.002 for trend), MACE, and the cardiorenal composite (Figure 1) although there were few events, and the increase was not statistically significant for MACE and the cardiorenal composite. In contrast, the incidence of the composite kidney end point increased 5-fold from HF stage A to HF stage B (pre-HF), albeit with few events and not statistically significant. The incidence of the composite kidney end point was comparable among participants with HF stage B and stage C/D (Figure 1). Results with stage B as the reference group are shown in Figure S1.

When participants with HF stage B were further disaggregated based on the presence of elevated biomarkers and echocardiographic abnormalities, the incidence of the primary outcomes was highest among participants with elevated biomarkers, particularly if both NT-proBNP and hs-cTnT were both elevated (Table S3). Likewise, when participants with HF stage B were separated into subgroups based on eGFR at baseline, the incidence of the primary outcome was highest among participants with lower eGFR (Table S4).

### Effect of Sotagliflozin versus Placebo According to HF Stage

**Figure 2.**
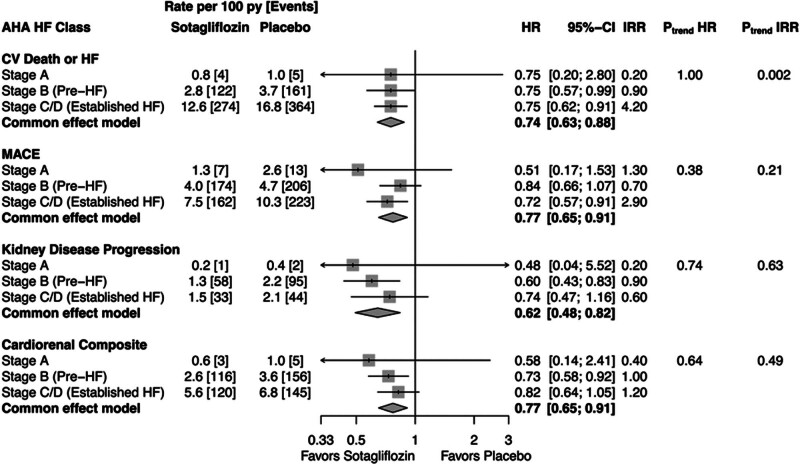
**Effect of sotagliflozin vs placebo on clinical outcomes stratified by 2022 heart failure (HF) stage.** MACE is a composite of cardiovascular (CV) death, nonfatal myocardial infarction, and nonfatal stroke. Kidney disease progression is a composite of ≥50% decline in estimated glomerular filtration rate (sustained or last value), kidney failure, or kidney death. Cardiorenal composite includes ≥50% decline in estimated glomerular filtration rate (sustained or last value), kidney failure, CV death, or kidney death. All hazard ratios (HRs) reflect stratification by randomization stratification factors. AHA indicates American Heart Association; IRR, incidence rate reduction; MACE, major cardiovascular events; and py, person-years.

For all outcomes examined, treatment with sotagliflozin versus placebo was associated with a consistent relative risk reduction across all HF stages (primary outcome: HR, 0.74 [95% CI, 0.63–0.88]; *P*_interaction_>0.5; Figure 2). Concordantly, the absolute risk reduction increased with HF stage for the primary outcome (*P*_trend_=0.002), but the trend for MACE and the cardiorenal composite did not achieve statistical significance. In contrast, the absolute risk reduction for the kidney-specific end point increased from HF stage A to HF stage B (pre-HF) and was comparable between HF stages B and C/D (*P*_trend_=0.63).

## Discussion

In this post hoc analysis of the SCORED trial, we found that the 2022 HF stages were associated with a higher risk of HF and MACE with advancing stages. Likewise, the incidence of the kidney-specific composite was 5-fold higher in stage B (pre-HF) versus stage A, but the incidence of kidney events was similar in stage B (pre-HF) and stage C/D (established HF). Sotagliflozin also had similar relative benefits for all end points compared with placebo, irrespective of HF stage, with a corresponding increase in absolute benefits at higher stages. The 2022 HF stages provide a parsimonious framework for risk stratification of cardiovascular and kidney outcomes with sotagliflozin demonstrating consistent efficacy across all stages.

Identifying participants who are at high risk of HF or CKD progression may guide the upstream use of SGLT inhibitors to prevent incident HF or progression to advanced CKD. The 2022 AHA classification was recently revised to leverage cardiac biomarkers to risk-stratify individuals with cardiovascular risk factors and identify those with abnormal biomarkers as being at increased risk for HF.^6^ These individuals are now classified as HF stage B and pre-HF. Our analysis demonstrated that only 7% of participants in SCORED were classified as stage A compared with 62% as stage B (pre-HF) and 31% as stage C/D. The percentage of adults with stage B (pre-HF) highlights the high prevalence of elevated cardiac biomarkers and echocardiographic abnormalities in those with diabetes and CKD. In analyses of the ARIC and Framingham prospective cohorts, the 2022 HF stages were also associated with an increased risk of HF and all-cause mortality,^12,14^ and participants classified as HF stage B solely based on cardiac biomarkers and in the absence of structural heart disease on echocardiogram were also at increased risk.^12,14^ Secondary analyses of randomized controlled trials of SGLT2 inhibitors have also documented associations between cardiovascular biomarkers and long-term cardiovascular events, across a range of patient populations, including individuals with and without established HF or CVD.^7,8,15^ Our analysis builds upon these findings, and we demonstrated that increasing HF stage among SCORED participants was associated with an increased risk of the primary composite outcome of death from cardiovascular causes, hospitalizations for HF, and urgent visits for HF.

Beyond HF, no studies have examined the association between HF stages and kidney outcomes. However, observational studies have documented associations between cardiac biomarkers and ESKD.^16–18^ As well, a secondary analysis of the CREDENCE trial (Canagliflozin and Renal Events in Diabetes With Established Nephropathy Clinical Evaluation) demonstrated that each unit increase in log-transformed NT-proBNP and hs-cTNT was associated with an increased risk of kidney composite of end-stage kidney disease, doubling of serum creatinine or kidney death.^8^ However, CREDENCE was restricted to participants with elevated albuminuria who were already at high risk of CKD progression. Therefore, the prognostic utility of cardiac biomarkers for kidney outcomes in a more generalizable CKD population remains to be established, particularly when biomarkers are operationalized alongside clinical and asymptomatic cardiac structural or functional abnormalities, such as in the 2022 HF classification.

In contrast to CREDENCE and other kidney outcome randomized controlled trials of SGLT inhibitors, SCORED did not include an albuminuria cutoff for eligibility, and many participants in SCORED were at moderate/intermediate risk for CKD progression based on the KDIGO (Kidney Disease: Improving Global Outcomes) risk criteria. This KDIGO risk category represents a highly heterogeneous population, and additional risk stratification tools are needed to distinguish participants who are at increased risk of CKD progression from those who are at low risk. Using the 2022 HF classification, our analysis demonstrated that the incidence of the composite kidney end point in placebo-treated participants in SCORED was only 0.4 per 100 person-years in HF stage A but increased 5-fold to 2.2 per 100 person-years in HF stage B (pre-HF) and 2.1 per 100 person-years in stage C/D. The marked increase in kidney risk from HF stage A to HF stage B (pre-HF) and the comparable risk for kidney outcomes in HF stage B (pre-HF) and stage C/D suggests that the 2022 HF classification appropriately disaggregates an intermediate kidney risk population into low versus high risk for CKD progression.

Finally, our results also highlight the shared pathophysiology and interdependent nature of pre-HF and established HF and CKD. For instance, the incidence of the primary outcome in HF stage B (pre-HF) was highest among participants with elevated biomarkers, particularly if both NT-proBNP and hs-cTnT were both elevated and in participants with the lowest eGFR at baseline. The effect of sotagliflozin was consistent across the 2022 HF stages and eGFR. The individual contributions of CKD and pre-HF toward long-term cardiorenal risk cannot be readily separated and instead highlight the importance of SGLT inhibitors as a medication class that show consistent benefit across patient characteristics, biomarkers, and disease phenotypes.

Given that the risk of CKD progression is already elevated in the earliest asymptomatic stages of HF, participants with stage B (pre-HF) may represent a subpopulation in whom kidney and cardioprotective medications may offer important joint benefits for reducing incident HF and CKD progression. Of note, our analysis also demonstrated that the relative risk reduction of sotagliflozin versus placebo for all outcomes was consistent across HF stages, with a corresponding increase in absolute benefits at higher stages. For kidney outcomes, the absolute benefit was comparable in participants with HF stage B (pre-HF) and stage C/D. These findings confirm the efficacy of sotagliflozin^9–11,19–22^ and other SGLT inhibitors across broad patient populations^23–25^ and reinforce the importance of their early use for primary prevention of HF and progression of CKD to advanced disease.

As participants in SCORED had diabetes and CKD at baseline, the baseline risk for cardiovascular and kidney outcomes in participants classified as HF stage A may be higher than in the general population in real-world studies. Therefore, the risk associated with HF stage B (pre-HF) or stage C/D may be underestimated in our study. For instance, in a study combining data from 3 longitudinal cohorts including the Multi-Ethnic Study of Atherosclerosis, the Cardiovascular Health Study, and the Framingham Heart Study, participants classified as HF stage B (pre-HF) had a 10-fold increased risk of incident HF (HR, 10.61 [95% CI, 9.0–12.51]) compared with participants classified as HF stage A. Likewise, in an analysis of the Atherosclerosis Risk in Communities, participants classified as HF stage B (pre-HF) had a 4-5-fold increased risk of incident HF (HR, 4.16 [95% CI, 2.78–6.21] for nondiabetes; HR, 5.32 [95% CI, 3.48–8.13] for diabetes) compared with participants classified as HF stage A. Therefore, our results regarding the risk associated with HF stages and the absolute benefit of sotagliflozin can be reasonably considered conservative.

Our results have important clinical and research implications. First, our findings support the routine measurement of cardiac biomarkers, and the use of the 2022 HF classification system risk stratifies participants for HF, MACE, and kidney outcomes. Our findings also guide early use of SGLT inhibitors and other kidney and cardioprotective medications in individuals who may otherwise be considered at low to intermediate risk. From a research standpoint, our findings suggest that individuals with HF stage B (pre-HF) are at high baseline risk for HF, MACE, and kidney outcomes and may be particularly suitable for enrollment in clinical trials focused on the primary prevention of HF and CKD. For instance, SGLT inhibitors have not been studied for HF or CKD prevention in adults with established CVD or multiple risk factors for HF, in the absence of diabetes or proteinuria. This patient population represents the last and arguably the largest unstudied population for the SGLT inhibitor. For this population, the 2022 AHA HF classification could be used to identify those with HF stage B (pre-HF) for a dedicated clinical trial on SGLT inhibitors. Specifically, the use of cardiac biomarkers such as NT-proBNP and hs-cTnT can be leveraged as risk enhancement criteria, which would increase the incidence of HF by up to 4-fold and, based on our study, likely increase the incidence of a kidney progression outcome by 2-fold. This would ultimately reduce sample size requirements and increase the feasibility for a dedicated clinical trial to expand the use of the SGLT inhibitor to this new population.

This study has important strengths. First, SCORED is unique as 99.5% of participants had biomarkers and LVEF measured at baseline, which is a higher percentage than other SGLT inhibitor clinical trials. For instance, the VERTIS-CV trial (Evaluation of Ertugliflozin Efficacy and Safety Cardiovascular Outcomes Trial) had LVEF measurements in only 60% of participants, and the CREDENCE trial had biomarker measurements in only 60% of participants.^8,26^ Second, SCORED did not include an albuminuria cutoff for eligibility and, therefore, includes participants across the full spectrum of kidney risk. This study also has important limitations. First, this was a post hoc analysis, and the findings are best viewed as hypothesis-generating. Nonetheless, we used established definitions to classify participants according to the 2022 HF stages and adhered to prespecified end points in SCORED. Second, the only diagnostic test to assess cardiac structure/function in SCORED was echocardiography, and the only parameters assessed were LVEF and left ventricular hypertrophy. We did not have information on parameters such as wall motion abnormalities, chamber enlargement, or established valvular disease. Therefore, there may be participants classified as having HF stage A who have other markers of structural heart disease, which were not captured in SCORED. These estimates of the risk associated with stage B (pre-HF) could, therefore, be considered conservative. Third, there were few kidney events, and this limited the statistical power to examine for differences by HF stage. Fourth, NT-proBNP and hs-cTnT may be elevated in individuals with CKD. However, baseline eGFR and UACR were comparable across all HF stages and are unlikely to result in differential misclassification of participants as HF stage B. Fifth, we lacked baseline data on severity of HF symptoms and frequency of HF hospitalizations to reliably distinguish between participants with HF stage C or D. Finally, SCORED included participants with at least moderate CKD at baseline, irrespective of UACR. Therefore, the findings of our analysis may not be generalizable to participants with milder forms of CKD.

In conclusion, increasing HF stage is associated with a higher risk of HF, MACE, and kidney events. Sotagliflozin provided consistent relative benefits for all end points, irrespective of HF stage, with a corresponding increase in absolute benefits at higher HF stages. These data highlighting the risk associated with stage B (pre-HF) should encourage physicians to stage HF risk among all patients with risk factors for HF, including hypertension, diabetes, and CKD.

## Article Information

### Acknowledgments

The authors thank all investigators, trial teams, and patients for their participation in the trial.

### Author Contributions

Drs Bhatt, Pitt, Steg, Cherney, Szarek, and Banks were responsible for the methodology and data curation. Drs Odutayo, Bhatt, Szarek, Cherney, and Davies and P. Banks were responsible for the formal analysis. Dr Odutayo, Sridhar, Cherney, and Davies wrote the original draft. All authors reviewed and edited the manuscript, gave final approval of the manuscript, made the decision to submit for publication, and agreed to be accountable for all aspects of the work in ensuring that questions related to the accuracy or integrity of any part of the work are appropriately investigated and resolved.

### Sources of Funding

The SCORED trial (Effect of Sotagliflozin on Cardiovascular and Renal Events in Patients With Type 2 Diabetes and Moderate Renal Impairment Who Are at Cardiovascular Risk) was funded by Lexicon Pharmaceuticals Inc (The Woodlands, TX), and Sanofi. The present analyses were funded by Lexicon Pharmaceuticals Inc. Dr Odutayo is supported by the Canadian Institutes of Health Research (CIHR) Research Excellence, Diversity, and Independence Early Career Transition Award, the Kidney Foundation of Canada, the Ted Rogers Center for Heart Research, and the University of Toronto Black Research Network. Dr Sridhar is supported by the Department of Medicine Eliot Phillipson Clinician-Scientist Training Program, a Banting and Best Diabetes Center Postdoctoral fellowship at the University of Toronto, and a CIHR Frederick Banting and Charles Best Canada Graduate Scholarships Doctoral Research Award. Dr Cherney is supported by the Department of Medicine, University of Toronto Merit Award and receives support from the CIHR, Diabetes Canada, and the Heart and Stroke Richard Lewar Center of Excellence. Dr Cherney is also the recipient of a CIHR-KFOC Teams Grant Award with additional funding from Breakthrough-T1D and is the Gabor Zellerman Chair in Nephrology Research, University of Toronto.

### Disclosures

Dr Odutayo reports the following disclosure: consultancy: Lexicon Pharmaceuticals and Novo Nordisk Canada. Dr Bhatt is the Chair of SCORED (Effect of Sotagliflozin on Cardiovascular and Renal Events in Patients With Type 2 Diabetes and Moderate Renal Impairment Who Are at Cardiovascular Risk) with research funding paid by Lexicon to Mount Sinai Hospital and discloses the following relationships: advisory board: AngioWave, Bayer, Boehringer Ingelheim, CellProthera, Cereno Scientific, E-Star Biotech, High Enroll, Janssen, Level Ex, McKinsey, Medscape Cardiology, Merck, NirvaMed, Novo Nordisk, Stasys, and Tourmaline Bio; board of directors: American Heart Association (AHA) New York City, AngioWave (stock options), Bristol Myers Squibb (stock), DRS.LINQ (stock options), and High Enroll (stock); consultant: Broadview Ventures, Corcept Therapeutics, GlaxoSmithKline, Hims, SFJ, Summa Therapeutics, and Youngene; data monitoring committees: Acesion Pharma, Assistance Publique-Hôpitaux de Paris, Baim Institute for Clinical Research (formerly Harvard Clinical Research Institute, for the PORTICO trial, funded by St. Jude Medical, now Abbott), Boston Scientific (Chair, PEITHO trial), Cleveland Clinic, Contego Medical (Chair, PERFORMANCE 2), Duke Clinical Research Institute, Mayo Clinic, Mount Sinai School of Medicine (for the ENVISAGE trial, funded by Daiichi Sankyo; for the ABILITY-DM trial, funded by Concept Medical; and for ALLAY-HF, funded by Alleviant Medical), Novartis, and Population Health Research Institute, Rutgers University (for the National Institutes of Health–funded MINT trial); honoraria: American College of Cardiology (Senior Associate Editor, Clinical Trials and News and ACC.org; Chair, ACC Accreditation Oversight Committee), Arnold and Porter Law Firm (work related to Sanofi/Bristol Myers Squibb clopidogrel litigation), Baim Institute for Clinical Research (formerly Harvard Clinical Research Institute; AEGIS-II executive committee funded by CSL Behring), Belvoir Publications (Editor-in-Chief, *Harvard Heart Letter*), Canadian Medical and Surgical Knowledge Translation Research Group (clinical trial steering committees), CSL Behring (AHA lecture), Cowen and Company, Duke Clinical Research Institute (clinical trial steering committees, including for the PRONOUNCE trial, funded by Ferring Pharmaceuticals), HMP Global (Editor-in-Chief, *Journal of Invasive Cardiology*), *Journal of the American College of Cardiology* (Guest Editor; Associate Editor), Level Ex, Medtelligence/ReachMD (CME steering committees), MJH Life Sciences, Oakstone CME (Course Director, Comprehensive Review of Interventional Cardiology), Piper Sandler, Population Health Research Institute (for the COMPASS operations committee, publications committee, steering committee, and USA national co-leader, funded by Bayer), WebMD (CME steering committees), and Wiley (steering committee); other: Clinical Cardiology (Deputy Editor); Patent: Sotagliflozin (named on a patent for sotagliflozin assigned to Brigham and Women’s Hospital who assigned to Lexicon; neither Dr Bhatt nor Brigham and Women’s Hospital receive any income from this patent); research funding: Abbott, Acesion Pharma, Afimmune, Aker Biomarine, Alnylam, Amarin, Amgen, AstraZeneca, Bayer, Beren, Boehringer Ingelheim, Boston Scientific, Bristol Myers Squibb, Cardax, CellProthera, Cereno Scientific, Chiesi, CinCor, Cleerly, CSL Behring, Faraday Pharmaceuticals, Ferring Pharmaceuticals, Fractyl, Garmin, HLS Therapeutics, Idorsia, Ironwood, Ischemix, Janssen, Javelin, Lexicon, Lilly, Medtronic, Merck, Moderna, MyoKardia, NirvaMed, Novartis, Novo Nordisk, Otsuka, Owkin, Pfizer, PhaseBio, PLx Pharma, Recardio, Regeneron, Reid Hoffman Foundation, Roche, Sanofi, Stasys, Synaptic, The Medicines Company, Youngene, and 89Bio; Royalties: Elsevier (Editor, Braunwald’s Heart Disease); and Site Co-Investigator: Cleerly. Dr Sridhar reports the following disclosures: conference and travel support from Merck Canada and Lexicon Pharmaceuticals; participation in the advisory board with Novo Nordisk Canada. Dr Szarek receives salary support from CPC, a nonprofit academic research organization affiliated with the University of Colorado, which receives or received research grant/consulting funding between July 2021 and July 2025 from the following organizations: 35Pharma Inc, Abbott Laboratories, Agios Pharmaceuticals Inc, Alexion Pharma Godo Kaisha, AHA, *American Journal of Managed Care*, Amgen Inc, Amgen USA Inc, Anthos Therapeutics Inc, Arrowhead Pharmaceuticals, AstraZeneca Pharma India, AstraZeneca Pharmaceuticals LP, AstraZeneca UK Ltd, Autonomy Bio Inc, Bayer, Bayer Aktiengesellschaft, Beth Israel Deaconess Medical Center, Better Therapeutics, Boston Clinical Research Institute LLC, Bristol Myers Squibb, Cleerly Inc, Clergy United for the Transformation of Sandtown, Colorado Department of Public Health and Environment, Congress Inc, Cook Regentec LLC, Eidos Therapeutics Inc, EluraBio Inc, Esperion Therapeutics Inc, Faraday Pharmaceuticals Inc, Gasherbrum Bio Inc, Insmed, IsomAb Limited, JanOne Biotech Holdings Inc, Janssen Global Services, Janssen Pharmaceuticals Inc, Janssen Scientific Affairs LLC, Las Animas and Huerfano Counties, District Health Department, Lexicon Pharmaceuticals Inc, Lilly USA LLC, Medison Pharma, Medpace Inc, Merck Sharp & Dohme Corp, Nectero Medical Inc, NewAmsterdam Pharma, Novartis Pharmaceuticals Corporation, Novo Nordisk Inc, Pfizer, Piper Sandler & Co, PPD Development L.P., Prothena Biosciences Limited, Regeneron, Regents of the University of Colorado (aka UCD), Sanifit Therapeutics S.A., Sanofi, Silence Therapeutics PLC, Stanford University, Stealth BioTherapeutics Inc, The Brigham and Women’s Hospital, Thrombosis Research Institute, Tourmaline Bio Inc, University of Colorado, University of Pittsburgh, VarmX, Verve Therapeutics, and WraSer LLC. Dr Szarek also reports serving as a consultant or research support (or both) from Amarin, Lexicon, NewAmsterdam, Novartis, Regeneron, Sanofi, Silence, and Tourmaline. Dr Cannon reports the following disclosures: research grants from Amgen, Better Therapeutics, Boehringer Ingelheim, Bristol Myers Squibb, Daiichi Sankyo, Janssen, Merck, Novo Nordisk, and Pfizer; received consulting fees from Aegerion/Amryt, Alnylam, Amarin, Amgen, Applied Therapeutics, Ascendia, Boehringer Ingelheim, Bristol Myers Squibb, Eli Lilly, Janssen, Lexicon, Merck, Pfizer, Rhoshan, and Sanofi; and has served on the Data and Safety Monitoring Boards for the Veterans Administration, Applied Therapeutics, and Novo Nordisk. Dr Leiter reports the following disclosures: research funding from Lexicon as a member of the study Executive Committee and received research funding from, has provided CME on behalf of, and has acted as an advisor to Abbott, Amgen, AstraZeneca, Bayer, Boehringer Ingelheim, Eli Lilly, GSK, HLS, Merck, Novartis, Novo Nordisk, Regeneron, Sanofi, and Servier. Dr McGuire reports the following disclosures: personal fees for consulting from Boehringer Ingelheim, Sanofi US, Merck & Co, Merck Sharp and Dohme Corp, Eli Lilly USA, Novo Nordisk, AstraZeneca, Lexicon Pharmaceuticals, Eisai, Pfizer, Metavant, Applied Therapeutics, Afimmune, Bayer, CSL Behring, and Esperion; received research support for Clinical Trials Leadership from Boehringer Ingelheim, Pfizer, AstraZeneca, Novo Nordisk, Esperion, Lilly USA, and CSL Behring; and received honoraria for consultancy from Lilly USA, Pfizer, Boehringer Ingelheim, Lexicon, Novo Nordisk, Applied Therapeutics, Altimmune, CSL Behring, Bayer, Intercept, and New Amsterdam. Dr Lewis reports the following disclosures: consultant fees from Sanofi. Dr Lopes reports the following disclosures: research grants or contracts from Amgen, Bristol Myers Squibb, GlaxoSmithKline, Medtronic, Pfizer, and Sanofi-Aventis; funding for educational activities or lectures from Pfizer, Daiichi Sankyo, and Novo Nordisk; and funding for consulting from Bayer, Boehringer Ingelheim, Bristol Myers Squibb, and Novo Nordisk. Dr Scirica reports the following disclosures: institutional research grants to Brigham and Women’s Hospital from Better Therapeutics, Merck, Novo Nordisk, and Pfizer; consulting fees from Allergan, Boehringer Ingelheim, Better Therapeutics, Elsevier PracticeUpdate Cardiology, Esperion, Hanmi, Lexicon, and Novo Nordisk; and equity in health at Scale and Doximity. Dr Ray reports the following disclosures: research grants from Amgen, Sanofi, Regeneron, Daiichi Sankyo, and Ultragenyx to Imperial College London; consulting fees from Novartis, Daiichi Sankyo, Kowa, Esperion, Novo Nordisk, MSD, Lilly, Silence Therapeutics, Az, New Amsterdam Pharma, Bayer, Beren Therapeutics, Cleerly, EmendoBio, Scribe, Crispr, Vaxxinity, Amarin, Regeneron, Ultragenyx, Sanofi Cargene, and Resverlogix; honoraria for lectures Novartis, Bi, AZ, Novo Nordisk, Viatris, Amarin, Biologix Pharma, Sanofi, Amgen, Esperion, Daiichi Sankyo, Mankind, and Macleod Pharma; and stock options from holding stock options from New Amsterdam Pharma, SCRIBE Therapeutics, and Pemi31. Dr Davies reports employment and stock/stock options from Lexicon Pharmaceuticals Inc. P. Banks reports employment and stock ownership from Lexicon Pharmaceuticals Inc. M. Girard reports consulting for Lexicon Pharmaceuticals Inc. Dr Verma reports the following disclosures: Tier 1 Canada Research Chair in Cardiovascular Surgery; receiving grants, and research support and speaking honoraria from Amarin, Amgen, AstraZeneca, Bayer, Boehringer Ingelheim, Canadian Medical and Surgical Knowledge Translation Research Group, Eli Lilly, HLS Therapeutics, Humber River Health, Janssen, Merck, Novartis, Novo Nordisk, Pfizer, PhaseBio, S & L Solutions Event Management Inc, Sanofi, and Sun Pharmaceuticals. He is the President of the Canadian Medical and Surgical Knowledge Translation Research Group, a federally incorporated not-for-profit physician organization. Dr Udell reports the following disclosures: advisory board: Boehringer Ingelheim, Novavax, Novo Nordisk, and Sanofi; speaker honoraria: Amgen, AstraZeneca, Boehringer Ingelheim, and Eli Lilly; and research funding to his institution: Amgen, Bayer, Boehringer Ingelheim, and Novartis. Dr Pitt reports the following disclosures: consultant: Lexicon, Bayer, Boehringer Ingelheim, AstraZeneca, Anacardio, Vifor, SC Pharmaceuticals, SQinnovations, G3 Pharmaceuticals, KBP Biosciences, Cereno Scientific, Sarfez Pharmaceuticals, Prointel, Sea Star Medical, and Brainstorm Medical; has stock/stock options in Vifor, SC Pharmaceuticals, SQinnovations, G3 Pharmaceuticals, KBP Biosciences, Cereno Scientific, Sarfez Pharmaceuticals, Prointel, Sea Star Medical, and Brainstorm Medical; and US Patent 99313412 site specific delivery of eplerenone to the myocardium; US patent pending 63/045,783 Histone Modulating agents for the prevention and treatment of organ damage. Dr Cherney reports the following disclosures: honoraria from Boehringer Ingelheim-Lilly, Merck, Bayer, AstraZeneca, Sanofi, Mitsubishi-Tanabe, AbbVie, Janssen, Bayer, Prometic, BMS, Maze, Gilead, CSL Behring, Otsuka, Novartis, Youngene, GSK, Amgen, Biobridge, Vantage, Altimmune, Lexicon, and Novo Nordisk; operational funding for clinical trials from Boehringer Ingelheim-Lilly, Merck, Janssen, Sanofi, AstraZeneca, CSL Behring, Novo Nordisk, Lexicon, and Bayer. Dr Steg reports no conflicts.

### Supplemental Material

Tables S1–S4

Figure S1

## Supplementary Material
