# Supplementary material for: Association Between the 2022 AHA/ACC/HFSA Heart Failure Staging and Cardiovascular and Kidney Outcomes in Patients With Diabetes and Kidney Disease: A Post Hoc Analysis of the SCORED Randomized Controlled Trial
Source: Circ Heart Fail. 2026 Jan 22;19(3):e013054. doi: 10.1161/CIRCHEARTFAILURE.125.013054 (PMC12986030; doi:10.1161/CIRCHEARTFAILURE.125.013054)
Supplement: Supplementary file 1 [file hhf-19-e013054-s001.pdf]

Supplemental Table 1: Availability of Data for Participants with Incomplete Data for Classification of Heart Failure Stage

History of HF	NT-proBNP<125	hs-cTnT<14	Left Ventricular Ejection Fraction	History of Left Ventricular Hypertrophy	Frequency of Missingness
No	Missing	Missing	≥50%	No	1
No	Missing	Yes	≥50%	No	1
No	Yes	Missing	≥50%	No	6
No	Yes	Yes	≥50%	Missing	43
No	Yes	Yes	Missing	Missing	2

Supplemental Table 2: Baseline Characteristics of Participants by Heart Failure Stage Using the 2022 Classification and Treatment Assignment

	Stage A		Stage B		Stage C/D	
	**Sotagliflozin (n=376)**	**Placebo (n=365)**	**Sotagliflozin (n=3276)**	**Placebo (n=3284)**	**Sotagliflozin (n=1640)**	**Placebo (n=1643)**
Age, years	66 (61, 71)	67 (60, 72)	69 (63, 74)	69 (63, 74)	70 (64, 75)	69 (64, 75)
Female	218 (58.0)	231 (63.3)	1418 (43.3)	1472 (44.8)	711 (43.4)	704 (42.8)
Race						
White	295 (78.5)	287 (78.6)	2676 (81.7)	2640 (80.4)	1431 (87.3)	1420 (86.4)
Black	19 (5.1)	24 (6.6)	114 (3.5)	110 (3.3)	43 (2.6)	54 (3.3)
Asian	31 (8.2)	25 (6.8)	188 (5.7)	238 (7.2)	98 (6.0)	102 (6.2)
American Indian or Alaska Native	16 (4.3)	17 (4.7)	163 (5.0)	174 (5.3)	27 (1.6)	25 (1.5)
Native Hawaiian or other Pacific Islander	4 (1.1)	0	12 (0.4)	11 (0.3)	9 (0.5)	4 (0.2)
Multiple	5 (1.3)	9 (2.5)	78 (2.4)	59 (1.8)	26 (1.6)	27 (1.6)
Unknown	6 (1.6)	3 (0.8)	45 (1.4)	52 (1.6)	6 (0.4)	11 (0.7)
Hispanic/Latino ethnicity	140 (37.2)	150 (41.1)	1200 (36.6)	1188 (36.2)	331 (20.2)	340 (20.7)
BMI, kg/m^2	31.6 (27.8, 35.9)	31.4 (28.0, 36.3)	31.8 (28.0, 36.0)	31.5 (27.7, 35.7)	32.0 (28.4, 36.6)	32.4 (28.7, 36.7)
SBP, mmHg	138 (125, 147)	136 (126, 146)	140 (129, 150)	140 (129, 150)	135 (124, 145)	135 (124, 146)
DBP, mmHg	80 (70, 88)	78 (71, 85)	78 (70, 85)	79 (70, 86)	77 (70, 84)	77 (69, 84)
Diabetes Duration, years	16.1 (9.6, 21.0)	14.5 (9.3, 20.0)	17.3 (10.6, 23.2)	17.0 (10.7, 22.7)	15.5 (9.9, 21.4)	15.2 (9.7, 21.6)
LVEF, %	62 (58, 67)	64 (60, 68)	60 (55, 65)	60.0 (55.0, 65.0)	50 (38, 60)	50 (37, 60)
A1C, %	8.1 (7.5, 9.1)	8.2 (7.5, 9.4)	8.3 (7.6, 9.3)	8.3 (7.6, 9.3)	8.4 (7.6, 9.5)	8.4 (7.6, 9.5)
NT-proBNP, pg/mL	52.0 (50.0, 82.3)	54.9 (50.0, 85.1)	169.5 (70.3, 401.9)	177.2 (67.5, 390.0)	471.7 (170.8, 1332.4	493.0 (155.0, 1370.3)
NT-proBNP ≥125 pg/mL	0 (0)	0 (0)	2020 (61.7)	2035 (62.0)	1330 (81.1)	1308 (79.6)
Hs-cTnT, pg/mL	9.0 (4.0, 11.7)	9.3 (4.0, 11.6)	18.9 (13.5, 27.3)	18.9 (13.3, 28.5)	23.0 (14.9, 37.8)	23.9 (15.4, 38.3)
Hs-cTnT ≥14 pg/mL	0 (0)	0 (0)	2390 (73.0)	2369 (72.1)	1277 (77.9)	1301 (79.2)
UACR, mg/g	35.4 (10.0, 207.5)	26.5 (8.8, 151.0)	97.3 (18.0, 636.6)	95.0 (19.0, 627.0)	55.0 (15.0, 320.0)	61.9 (14.0, 338.0)
eGFR, ml/min	49.8 (42.9, 54.6)	49.4 (43.1, 54.4)	44.4 (36.9, 51.2)	44.5 (36.7, 51.4)	43.4 (36.3, 50.4)	43.9 (36.5, 50.8)
KDIGO Risk Category						
Low Risk	13 (3.5)	7 (1.9)	59 (1.8)	48 (1.5)	22 (1.3)	19 (1.2)
Moderate Risk	121 (32.2)	135 (37.0)	559 (17.1)	595 (18.1)	305 (18.6)	341 (20.8)
High Risk	127 (33.8)	126 (34.5)	933 (28.5)	946 (28.8)	532 (32.4)	495 (30.1)
Very High Risk	115 (30.6)	97 (26.6)	1725 (52.7)	1695 (51.6)	781 (47.6)	788 (48.0)
Left Ventricular Hypertrophy	0 (0)	0 (0)	177 (5.4)	194 (5.9)	112 (6.8)	133 (8.1)
History of CVD	114 (30.3)	103 (28.2)	1427 (43.6)	1394 (42.4)	1036 (63.2)	1070 (65.1)
Myocardial Infarction	31 (8.2)	31 (8.5)	483 (14.7)	506 (15.4)	537 (32.7)	520 (31.6)

Stroke	24 (6.4)	14 (3.8)	261 (8.0)	284 (8.6)	187 (11.4)	176 (10.7)
Coronary Revascularization	38 (10.1)	38 (10.4)	619 (18.9)	605 (18.4)	551 (33.6)	524 (31.9)
Peripheral Vascular Disease	52 (13.8)	55 (15.1)	672 (20.5)	602 (18.3)	417 (25.4)	433 (26.4)
Medications						
RAASi	324 (86.2)	311 (85.2)	2900 (88.5)	2875 (87.5)	1481 (90.3)	1474 (89.7)
Loop diuretics	41 (10.9)	41 (11.2)	832 (25.4)	818 (24.9)	996 (60.7)	1008 (61.4)
Beta Blocker	139 (37.0)	149 (40.8)	1813 (55.3)	1809 (55.1)	1358 (82.8)	1348 (82.0)
Statins	274 (72.9)	268 (73.4)	2459 (75.1)	2368 (72.1)	1299 (79.2)	1328 (80.8)
Antiplatelets	191 (50.8)	182 (49.9)	1862 (56.8)	1855 (56.5)	1054 (64.3)	1073 (65.3)

Note: Values in table are median (Q1, Q3) or n (%)

Supplemental Table 3: Incidence of Cardiovascular Death and Heart Failure by Heart Failure Stage in the Placebo Group

Outcome	Stage A	Stage B				Stage C/D
		Normal NT-proBNP ***and*** hs-cTnT, Abnormal Echocardiogram	Elevated NT-proBNP ***or*** hs-cTnT, Normal Echocardiogram	Elevated NT-proBNP ***and*** hs-cTnT, Normal Echocardiogram	Elevated NT-proBNP and/or hs-cTnT and Abnormal Echocardiogram	
	N=741, (7%)	N= 618, (5.8%)	N=1395, (13.2%)	N=1534, (14.5%)	N=3013, (28.5%)	N= 3283, (31.0%)
CV Death and HF, N (rate per 100 patient years)	5 (1.01)	1 (0.232)	14 (1.44)	49 (4.78)	97 (4.92)	364 (16.83)

Supplemental Table 4: Incidence of Cardiovascular Death and Heart Failure in Participants Classified as Stage B Pre-HF by Baseline Estimated Glomerular Filtration Rate

Baseline eGFR (ml/min/1.73m2)	Placebo	Sotagliflozin	HR (95% CI)
	CV Death and HF, N (rate per 100 patient years)		
≥45	59 (2.76)	43 (2.04)	0.71 (0.45-1.13)
≥30 to <45	80 (4.12)	64 (3.32)	0.77 (0.53-1.14)
<30	22 (6.78)	15 (4.16)	0.55 (0.25-1.24)

eGFR is glomerular filtration rate; CV is cardiovascular; HF is heart failure; HR is hazard ratio; CI is confidence interval

Supplemental Figure 1: Association of the 2022 Heart Failure Stage with Clinical Outcome with Heart Failure Stage B as the Reference Group

AHA HF Class	Placebo Rate/100py [Events]	aHR	95%–CI
**CV Death or HF**			
Stage A	1.0 [5]	0.51	[0.21; 1.24]
Stage B (Pre–HF)	3.7 [161]	Reference	
Stage C/D (Established HF)	16.8 [364]	2.05	[1.55; 2.72]
**MACE**			
Stage A	2.6 [13]	0.84	[0.44; 1.60]
Stage B (Pre–HF)	4.7 [206]	Reference	
Stage C/D (Established HF)	10.3 [223]	1.31	[0.98; 1.75]
**Kidney Disease Progression**			
Stage A	0.4 [2]	0.47	[0.12; 1.88]
Stage B (Pre–HF)	2.2 [95]	Reference	
Stage C/D (Established HF)	2.1 [44]	1.26	[0.73; 2.16]
**Cardiorenal Composite**			
Stage A	1.0 [5]	0.59	[0.24; 1.45]
Stage B (Pre–HF)	3.6 [156]	Reference	
Stage C/D (Established HF)	6.8 [145]	1.42	[1.03; 1.95]

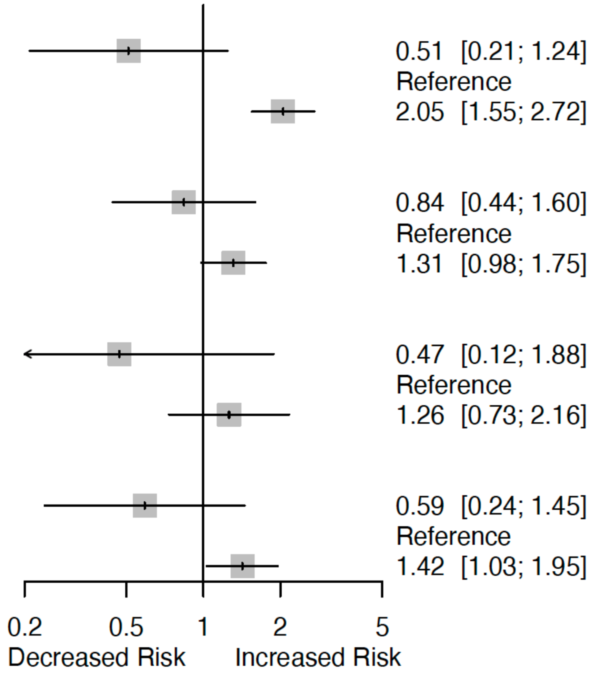

aHR is adjusted hazard ratio; CV is cardiovascular; HF is heart failure; py is person years; MACE is major cardiovascular events and is a composite of cardiovascular death, non-fatal myocardial infarction, and non-fatal stroke. Kidney disease progression is a composite of ≥50% decline in estimated glomerular filtration rate (sustained or last value), kidney failure, or kidney death. Cardiorenal composite includes ≥50% decline in estimated glomerular filtration rate (sustained or last value), kidney failure, cardiovascular death, or kidney death. All HRs are adjusted for age, sex, race, BMI, SBP, DBP, diabetes duration, HbA1c, eGFR, UACR, history of CVD (MI, stroke, coronary revascularization, or PVD), and concomitant medications (RAASi, loop diuretic, beta blocker, statin, and antiplatelet)
